# Patient-Physician Language Concordance and Cardiovascular Outcomes Among Patients With Hypertension

**DOI:** 10.1001/jamanetworkopen.2024.60551

**Published:** 2025-02-19

**Authors:** Michael Reaume, Mathieu N. Labossière, Ricardo Batista, Stephanie Van Haute, Navdeep Tangri, Claudio Rigatto, Clara Bohm, Denis Prud’homme, Peter Tanuseputro, Lisa M. Lix

**Affiliations:** 1 Department of Medicine, Faculty of Medicine, University of Ottawa, Ottawa, Ontario, Canada; 2 Department of Département de Médecine, Faculté de Médecine et des Sciences de la Santé, Université de Sherbrooke, Sherbrooke, Canada; 3 Akausivik Inuit Family Health Team, Ottawa, Ontario, Canada; 4 Ottawa Hospital Research Institute, Ottawa, Ontario, Canada; 5 College of Nursing, Max Rady College of Medicine, University of Manitoba, Winnipeg, Manitoba, Canada; 6 Manitoba Métis Federation, Winnipeg, Manitoba, Canada; 7 Chronic Disease Innovation Centre, Seven Oaks General Hospital, Winnipeg, Manitoba, Canada; 8 Université de Moncton, Moncton, New Brunswick, Canada; 9 Institut du Savoir Montfort, Ottawa, Ontario, Canada; 10 Department of Family Medicine and Primary Care, The University of Hong Kong, Pok Fu Lam, Hong Kong; 11 Department of Community Health Sciences, Max Rady College of Medicine, Winnipeg, Manitoba, Canada; 12 George and Fay Yee Centre for Healthcare Innovation, Winnipeg, Manitoba, Canada

## Abstract

**Importance:**

Patients who live in minority language communities often receive health care services of lower quality and safety compared with patients who speak the majority language. Yet the outcomes associated with care provided by physicians who speak a patient’s primary language remain unknown.

**Objective:**

To examine patient-physician language concordance and the risk of major adverse cardiovascular events (MACEs) among patients with hypertension.

**Design, Setting, and Participants:**

This retrospective cohort study identified adults with self-reported hypertension in the Canadian Community Health Survey, a national survey that collects data from a representative sample of Canadians, from January 1, 2003, to December 31, 2014. Respondents (excluding those living in Quebec) had their hospitalization and mortality records linked to their survey responses. Data were analyzed from October 2023 to May 2024.

**Exposures:**

Respondents’ primary home language was defined using language spoken most often at home. Language spoken with a regular physician was used to measure patient-physician language concordance. Respondents who spoke to their regular physician in their primary home language were classified as having received language-concordant care, while all other respondents were classified as having received language-discordant care.

**Main Outcomes and Measures:**

MACEs within 5 years of survey completion.

**Results:**

Among the 124 583 patients included in this study, 114 239 (91.7%) spoke English, 4790 (3.8%) spoke French, 325 (0.3%) spoke an Indigenous language, and 5229 (4.2%) spoke an allophone (ie, other) language. The mean (SD) age of the cohort was 63.7 (14.8) years; 57.1% of the patients reported their sex as female. Very few respondents who spoke an Indigenous language at home (<4.6%) received language-concordant care. For French-speaking patients, there was no statistically significant difference in the risk of MACE between those who received language-concordant care and those who received language-discordant care (hazard ratio [HR], 1.09; 95% CI, 0.86-1.36). Allophone-speaking patients who received language-concordant care were 36% less likely to experience MACE (HR, 0.64; 95% CI, 0.51–0.80) compared with allophone-speaking patients who received language-discordant care.

**Conclusions and Relevance:**

This retrospective cohort study found large disparities in both access to language-concordant care and risk of MACEs. These findings suggest that language-concordant care could potentially improve the health of individuals in minority language communities.

## Key Points

**Question:**

Is patient-physician language concordance associated with cardiovascular outcomes among patients with hypertension living in minority language communities?

**Findings:**

In this cohort study of 124 583 patients with hypertension in Canada, 5229 (4.2%) who primarily spoke an allophone language (ie, language other than English, French, or an Indigenous language) were identified. Allophones experienced 36% fewer major adverse cardiovascular events when they received care from physicians who spoke their primary home language compared with those who received care from physicians who did not speak their primary home language.

**Meaning:**

The findings of this study suggest that patient-physician language concordance is associated with a lower risk of cardiovascular outcomes among patients with hypertension.

## Teaser

This cohort study examines language concordance among patients and physicians in patients with hypertension living in minority language communities in Canada.

## Introduction

Hypertension is one of the most important modifiable risk factors for cardiovascular disease^1,2^ and one of the leading causes of morbidity and mortality across the world.^3^ While the age-adjusted prevalence of hypertension has remained stable over the past 3 decades, the absolute number of persons with hypertension has doubled.^4^ Hypertension affects approximately one-third of the world’s adult population^4^ and nearly half of all adults living in the US.^5,6^ Despite global public health initiatives aimed at improving early detection and treatment of hypertension,^7,8,9^ the prevalence of poorly controlled hypertension remains high.^5,6,7,8^ It is therefore not surprising that hypertension is one of the most common reasons for visiting a physician in a primary care setting^10^ and is responsible for approximately 10% of direct health care costs worldwide.^11,12^

As linguistic diversity across North America continues to increase,^13,14^ an increasing proportion of the population is at risk of experiencing poor health outcomes as a result of living in a minority language community, which occurs when an individual’s preferred language is not spoken by most residents in the region in which they live.^14^ Several studies have highlighted the role of language as a social determinant of health in the management of chronic diseases.^15,16,17,18^ For instance, studies conducted in the US have reported that non–English-speaking patients with diabetes, hypertension, and dyslipidemia who receive primary care from physicians who speak their preferred language (ie, language-concordant care) have better glycemic,^19,20,21^ blood pressure,^19^ and low-density lipoprotein cholesterol control^19,21^ compared with patients who received care communicated only in English (ie, language-discordant care). However, none of these studies was specifically designed or powered to assess patient-physician language concordance in terms of end-organ complications of chronic diseases. Patient-physician language discordance is potentially modifiable via, for example, referral of patients to physicians with proficiency in their preferred language or use of interpretation services, which can decrease the risk of adverse outcomes in situations of patient-physician language discordance when linguistic matching is not possible.^22,23^ Further investigation of this important risk factor is warranted.

The objective of this study was to examine patient-physician language concordance and the risk of major adverse cardiovascular events (MACEs) among a cohort of Canadians with hypertension. We hypothesized that patients with hypertension who received language-concordant care would have better outcomes compared with patients with hypertension who received language-discordant care.

## Methods

### Study Design and Population

We conducted a population-based retrospective cohort study using the Canadian Community Health Survey (CCHS).^24^ This cohort consisted of individuals older than 18 years with self-reported hypertension who were included in one of the annual cycles of the CCHS from January 1, 2003, to December 31, 2014.^25^ The cohort was then linked to administrative data to identify disease-specific outcomes. This study follows the Strengthening the Reporting of Observational Studies in Epidemiology (STROBE) reporting guideline for observational studies.^26^ The microdata files from the CCHS are made publicly available through a mechanism set out by legislation and regulation.^24,27^ As such, ethics approval was not required for their use (as outlined by the Tri-Council Policy Statement, article 2.2).^28^

### Setting

Canada has a population of nearly 37 million people living in 10 provinces and 3 territories.^29^ The two languages spoken most frequently are English and French, with 92.9% of the population speaking one of these two languages at home according to the 2021 census.^30^ English is the language spoken most often at home by 57.1% of Canadians, while French is the language spoken most often at home by 17.6% of Canadians.^30^ Both English and French are recognized as official languages at the federal level; New Brunswick is the only province where both English and French have official language status in all branches of the provincial government.^14^ French is the official language in Quebec, and English is the official language in the remaining provinces.^14^ The territories recognize English, French, and several Indigenous languages as official languages (eTable 1 in Supplement 1).^14^

### Data Sources

The CCHS is an annual cross-sectional survey conducted by Statistics Canada that collects health information for the Canadian population.^24^ The CCHS uses a standardized questionnaire to collect information on social determinants of health, health status, and health care use.^24^ Additional information regarding sampling frame, survey design, and survey weights are presented in the eMethods in Supplement 1.

Statistics Canada has linked the CCHS to several national databases, including the Canadian Vital Statistics Database (CVSD) and the Discharge Abstract Database (DAD).^27^ During CCHS assessments, interviewers ask respondents to provide consent for record linkage, which is then performed using either probabilistic record linkage (CVSD) or deterministic record linkage (DAD).^27^ The linkage rate for the CCHS cycles used in this study was 95.1%. Respondents who could not be successfully linked to both CVSD and DAD were excluded. The CVSD collects medical information (including cause of death) on all deaths, while the DAD collects data on admissions to acute care treatment facilities.^27^ The DAD captures hospitalizations for all provinces and territories except for Quebec, while hospitalizations in Manitoba have only been recorded in the DAD since April 1, 2004.^31^ For this reason, we excluded CCHS respondents living in Quebec and those living in Manitoba before April 1, 2004. Respondents were followed up until December 31, 2017.

### Exposure

We defined respondents’ primary home language using language spoken most often at home. We created 4 mutually exclusive linguistic groups: English only (anglophone), French only (francophone), Indigenous language only, and other language only (allophone). We restricted the group of Indigenous-speaking respondents to those who self-identified as First Nations, Inuit, or Métis, as these are 3 groups of Indigenous peoples recognized by the Canadian Constitution.^32^ For some respondents, language spoken most often at home did not correspond to one of the languages collected in the CCHS and was therefore coded as other. We classified these respondents as Indigenous speaking if they identified as First Nations, Inuit, or Métis, or as allophone speaking if they reported having immigrated to Canada. Respondents who spoke multiple languages at home were excluded if two or more of their languages corresponded to different linguistic groups (eg, English, French, Indigenous, and allophone), as these respondents could not be confidently assigned to a single linguistic group. We recognize the importance of avoiding pan-Indigenous language to describe the unique culture, linguistic, and historical experiences of different tribal groups; however, due to limitations around sample size, we made a decision to incorporate all Indigenous languages into a single category. A complete list of individual languages collected in the CCHS is presented in eTable 2 in Supplement 1.

Patient-physician language concordance was defined as concordance between the respondents’ primary home language and the language spoken with their regular physician. Respondents were said to have received language-concordant care if the language spoken with their regular physician corresponded to their linguistic group. Indigenous-speaking and allophone-speaking patients who spoke to their regular physician in a language coded as other were also considered to have received language-concordant care. All other respondents were said to have received language-discordant care.

### Covariates

We obtained the following baseline covariates from the CCHS: age, sex (male, female), marital status (single, married or common law), educational level (less than high school, high school graduate, postsecondary graduate), household income (quintiles 1 [lowest] to 5 [highest]), geographic region (western provinces/territories, Ontario, Maritime provinces), urban or rural residence, Indigenous identity, immigrant status, self-reported knowledge of English, diabetes, history of heart disease, obesity (body mass index <25.0, 25.0-29.9, ≥30.0, calculated as weight in kilograms divided by height in meters squared), smoking (daily or occasionally, former, never), and history of stroke.

### Outcomes

The primary outcome was first occurrence of MACEs within 5 years of survey completion. MACE was defined as acute coronary syndrome hospitalization, heart failure hospitalization, stroke hospitalization, or cardiovascular death. Secondary outcomes included individual components of the primary outcome, as well as all-cause hospitalization and all-cause death. Outcomes were identified through diagnostic codes, which have previously been validated to identify MACEs through administrative data.^33,34^ Definitions of outcomes, with corresponding diagnostic codes, are presented in eTable 3 in Supplement 1.

### Statistical Analysis

Data analysis was conducted from October 2023 to May 2024. We censored respondents at 5 years from CCHS assessments to minimize bias related to changes in health status during the follow-up period and/or misclassification with respect to the language spoken with the regular physician. Some patients may have had a different regular physician during the follow-up period, which could have resulted in exposure to both language-concordant and language-discordant care, depending on the linguistic abilities of the physician.

We report patient characteristics for each linguistic group (mean [SD] for continuous variables and number [percentage] for categorical variables). Outcomes for patients receiving language-concordant and language-discordant care are reported as percentages and were compared using the log-rank test. To comply with Statistics Canada policy on confidentiality and privacy,^35^ categorical variables with small sample sizes (n <15) are not reported due to risk of identifying individual respondents.

Multivariable Cox proportional hazards regression models were used to assess patient-physician language concordance and the primary and secondary outcomes. We planned to run separate regression models for each linguistic group representing a minority language community (ie, 1 for francophone, 1 for Indigenous, and 1 for allophone cohorts), but we could only perform analyses for the francophone and allophone groups (ie, 2 regression models) owing to the small number of Indigenous-speaking respondents who received care in an Indigenous language. Patients receiving language-discordant care were the reference group. Models were adjusted for age, sex, marital status, educational level, household income, geographic region, urban or rural residence, immigration status, history of cardiovascular disease (defined as history of heart disease or stroke), diabetes, obesity, and smoking. We used a cause-specific hazard model to adjust for the competing risk of death (or noncardiovascular death, where applicable). Proportional hazards assumption was assessed by visual inspection of the Kaplan-Meier survival curves for each of the primary and secondary outcomes. We performed multiple imputations by chained equations to address missing data (eTable 4 in Supplement 1).

We tested for interactions between patient-physician language concordance and prespecified subgroups of interest. To do so, we ran multivariable Cox proportional hazards regression models for each baseline characteristic of interest with an additional term denoting the interaction between patient-physician language concordance and the given baseline characteristic. We then compared the extended model (including the interaction term) and complete model (without the interaction term) using a multivariate Wald test.^36^

Adjusted hazard ratios (HRs) and their 95% CIs were estimated. Statistical tests were 2-tailed, and the significance threshold was set at *P* < .05. All analyses were performed using R software, version 4.2.3 (R Foundation for Statistical Computing).^37^

## Results

**Figure 1.**
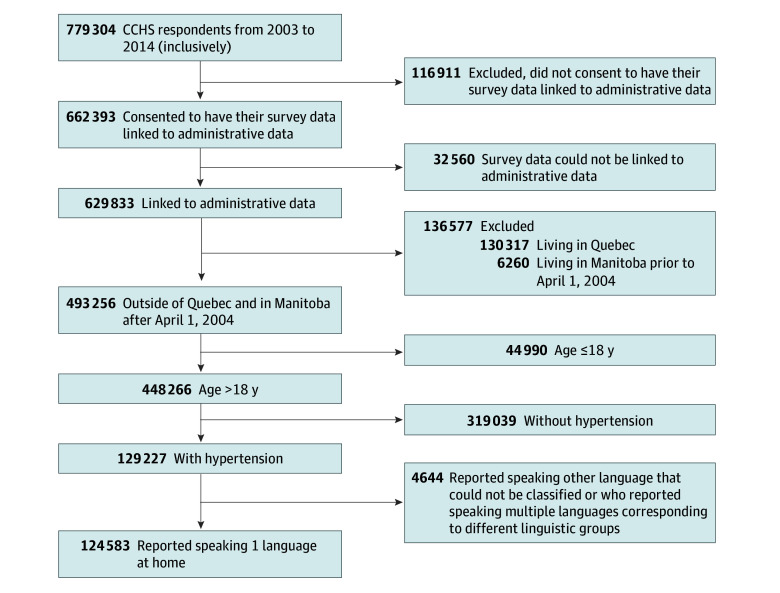
Study Flow Diagram CCHS indicates Canadian Community Health Survey.

We studied 124 583 patients with self-reported hypertension (Figure 1). The mean (SD) age of the cohort was 63.7 (14.8) years; 57.1% of the patients reported their sex as female. Complete baseline characteristics of the cohort are presented in Table 1.

**Table 1.** Baseline Characteristics of Patients With Hypertension, Stratified by Linguistic Group

Baseline characteristic	Linguistic group, No. (%)			
	Anglophone (n = 114 239)	Francophone (n = 4790)	Indigenous (n = 325)	Allophone (n = 5229)
Sociodemographic				
Age, mean (SD), y	63.7 (14.8)	63.5 (13.9)	52.9 (18.4)	63.6 (15.2)
Age range, y				
<60	40 300 (35.3)	1738 (36.3)	187 (57.5)	1843 (35.2)
60-75	45 103 (39.5)	1966 (41.0)	95 (29.2)	2002 (38.3)
≥75	28 836 (25.2)	1086 (22.7)	43 (13.2)	1384 (26.5)
Sex				
Female	65 178 (57.1)	2894 (60.4)	199 (61.2)	2914 (55.7)
Male	49 061 (42.9)	1896 (39.6)	126 (38.8)	2315 (44.3)
Marital status				
Single	49 824 (43.6)	2020 (42.2)	165 (50.8)	1573 (30.1)
Married or common law	64 310 (56.3)	2762 (57.7)	160 (49.2)	3653 (69.9)
Missing	105 (0.1)	8 (0.2)	0	3 (0.1)
Educational level				
Less than high school	31 519 (27.6)	2184 (45.6)	216 (66.5)	2034 (38.9)
High school graduate	27 101 (23.7)	696 (14.5)	41 (12.6)	872 (16.7)
Postsecondary graduate	54 576 (47.8)	1853 (38.7)	62 (19.1)	2252 (43.1)
Missing	1043 (0.9)	57 (1.2)	6 (1.8)	71 (1.4)
Household income quintile				
1 (Lowest)	26 385 (23.1)	1474 (30.8)	NA^a^	2068 (39.5)
2	24 370 (21.3)	1120 (23.4)	NA^a^	1130 (21.6)
3	20 252 (17.7)	724 (15.1)	NA^a^	669 (12.8)
4	16 985 (14.9)	625 (13.0)	NA^a^	456 (8.7)
5 (Highest)	16 380 (14.3)	487 (10.2)	NA^a^	296 (5.7)
Missing	9867 (8.6)	360 (7.5)	NA^a^	610 (11.7)
Geographic region				
Western provinces/territories	46 552 (40.7)	324 (6.8)	≥297 (≥91.4)^b^	2174 (41.6)
Ontario	46 372 (40.6)	1644 (34.3)	<15 (<4.6)^b^	2975 (56.9)
Maritime provinces	21 315 (18.7)	2822 (58.9)	<15 (<4.6)^b^	80 (1.5)
Urban/rural residence				
Rural	34 097 (29.8)	2183 (45.6)	181 (55.7)	465 (8.9)
Urban	80 142 (70.2)	2607 (54.4)	144 (44.3)	4764 (91.1)
Indigenous identity				
Yes	4835 (4.2)	180 (3.8)	325 (100.0)	<15 (<0.3)^b^
No	104 838 (91.8)	4575 (95.5)	0	≥3990 (≥76.3)^b^
Missing	4566 (4.0)	35 (0.7)	0	1225 (23.4)
Immigrant				
Yes	14 788 (12.9)	89 (1.9)	0	4705 (90.0)
No	99 272 (86.9)	4699 (98.1)	325 (100.0)	481 (9.2)
Missing	179 (0.2)	2 (<.01)	0	43 (0.8)
Knowledge of English (self-reported)^c^				
Yes	NR^d^	3729 (77.8)	282 (86.8)	4203 (80.4)
No	NR^d^	1060 (22.1)	43 (13.2)	1023 (19.6)
Missing	NR^d^	1 (<.01)	0	3 (0.1)
Comorbidity				
Diabetes	22 087 (19.3)	919 (19.2)	69 (21.2)	1108 (21.2)
History of heart disease	19 603 (17.2)	847 (17.7)	49 (15.1)	808 (15.5)
Obesity (BMI)				
Normal (<25.0)	31 282 (27.4)	1366 (28.5)	67 (20.6)	1775 (33.9)
Overweight (25.0-29.9)	41 942 (36.7)	1803 (37.6)	102 (31.4)	1940 (37.1)
Obese (≥30.0)	35 501 (31.1)	1413 (29.5)	101 (31.1)	1086 (20.8)
Missing	5514 (4.8)	208 (4.3)	55 (16.9)	428 (8.2)
Smoking				
Current	20 421 (17.9)	770 (16.1)	169 (52.0)	469 (9.0)
Former	60 552 (53.0)	2562 (53.5)	113 (34.8)	1685 (32.2)
Never	32 960 (28.9)	1447 (30.2)	41 (12.6)	3062 (58.6)
Missing	306 (0.3)	11 (0.2)	2 (0.6)	13 (0.2)
History of stroke	4755 (4.2)	192 (4.0)	19 (5.8)	250 (4.8)
Health care interactions				
Regular physician				
Yes	107 497 (94.1)	4569 (95.4)	133 (40.9)	5013 (95.9)
No	6703 (5.9)	220 (4.6)	192 (59.1)	216 (4.1)
Missing	39 (<.01)	1 (<.01)	0	0
Patient-physician language concordance^e^				
Yes	107 097 (99.6)	3134 (68.6)	<15 (<4.6)^b^	1708 (34.1)
No	383 (0.4)	1432 (31.3)	≥118 (≥36.3)^b^	3305 (65.9)
Missing	17 (<.01)	3 (0.1)	193 (59.4)	0

Abbreviations: BMI, body mass index (calculated as weight in kilograms divided by height in meters squared); NA, not asked; NR, not relevant.

^a^ Questions pertaining to household income were not asked of respondents living in the territories.

^b^ Categorical variables with small sample sizes (n <15) were not reported due to risk of identifying individual respondents.

^c^ Variable derived from question that asks respondents whether they speak English and French well enough to conduct a conversation in each language. Answer choices include English only, French only, both English and French, and neither English nor French. Respondents who answered English only or both English and French were considered to have knowledge of English, while those who answered French only and neither English nor French were considered to not have knowledge of English.

^d^ Variable not relevant for respondents whose primary home language is English.

^e^ Denotes the percentage of respondents compared with those who have a regular physician.

A total of 114 239 individuals were in the anglophone cohort (91.7%), 4790 in the francophone cohort (3.8%), 325 in the Indigenous cohort (0.3%), and 5229 in the allophone (4.2%) cohort. A complete list of individual Indigenous and allophone languages represented in this study, with frequencies corresponding to each language, is presented in eTable 5 and eTable 6 in Supplement 1. The mean (SD) age was lower among respondents who spoke an Indigenous language (52.9 [18.4] years) compared with the anglophone (63.7 [14.8] years), francophone (63.5 [13.9] years), and allophone (63.6 [15.2] years) cohorts. Patients whose primary home language was a language other than English had lower levels of education (45.6% of francophones [2184 of 4790] and 38.9% of allophones [2034 of 5229] had not completed high school) and income (30.8% of francophones [1474 of 4790] and 39.5% of allophones [2068 of 5229] were in the lowest household income quintile) compared with those in the anglophone group (only 27.6% had not completed high school [31 519 of 114 239] and only 23.1% were in the lowest household income quintile [26 385 of 114 239]). Most allophone-speaking patients (90.0% [4705 of 5229]) immigrated to Canada. Self-reported knowledge of English was similar across the 3 nonanglophone linguistic groups (77.8% for French-speaking patients [3729 of 4790]; 86.8% for Indigenous-speaking patients [282 of 325]; and 80.4% for allophone-speaking patients [4203 of 5229]).

Individuals in the anglophone, francophone, and allophone groups were equally likely to have a regular physician (anglophone, 94.1% [107 497 of 114 239]; francophone, 95.4% [4569 4790]; and allophone, 95.9% [5013 of 5229]), while less than half of the respondents who spoke an Indigenous language at home (40.9% [133 of 325]) had a regular physician. Nearly all those in the anglophone cohort (99.6% [107 097 of 114 239]) spoke to their physician in English, while 68.6% of the patients in the francophone cohort (3134 of 4790) and 34.1% of those in the allophone cohort (1708 of 5229) received language-concordant care. Very few respondents who spoke an Indigenous language at home (<4.6%) received language-concordant care. Baseline characteristics of the francophone and allophone cohorts stratified by patient-physician language concordance are presented in eTable 7 in Supplement 1.

### Primary Outcome: MACEs

The percentage of individuals in the francophone cohort who experienced MACEs did not differ significantly comparing those who received language-concordant care with those who received language-discordant care in the unadjusted analysis (8.7% [272 of 3134] vs 9.1% [130 of 1432]; *P* = .62). Patients in the allophone group who received language-concordant care were less likely to experience MACEs compared with those in the allophone group who received language-discordant care in the unadjusted analysis (6.5% [111 of 1708] vs 9.1% [301 of 3305]; *P* < .001). Complete results for the unadjusted analysis of secondary outcomes stratified by patient-physician language concordance are presented in Table 2.

**Table 2.** Unadjusted Outcomes for Patients With Hypertension, Stratified by Patient-Physician Language Concordance^a^

Baseline characteristic	Patients, No. (%)					
	Francophone cohort (n = 4566)			Allophone cohort (n = 5013)		
	Language-concordant (n = 3134)	Language-discordant (n = 1432)	*P* value	Language-concordant (n = 1708)	Language-discordant (n = 3305)	*P* value
Primary outcome, MACEs^b^	272 (8.7)	130 (9.1)	.62	111 (6.5)	301 (9.1)	<.001
Secondary outcomes						
Acute coronary syndrome^b^	139 (4.4)	61 (4.3)	.82	36 (2.1)	125 (3.8)	<.001
Heart failure	70 (2.2)	42 (2.9)	.15	31 (1.8)	91 (2.8)	.04
Stroke	57 (1.8)	30 (2.1)	.51	40 (2.3)	71 (2.1)	.72
Cardiovascular death	77 (2.5)	47 (3.3)	.11	45 (2.6)	121 (3.7)	.05
Hospitalization (all cause)	1322 (42.2)	611 (42.7)	.47	527 (30.9)	1292 (39.1)	<.001
Death (all cause)	253 (8.1)	138 (9.6)	.08	135 (7.9)	322 (9.7)	.03

Abbreviation: MACEs, major adverse cardiovascular events.

^a^ Unadjusted outcomes could not be reported for Indigenous-speaking patients due to risk of identifying individual respondents with small sample sizes (n <15).

^b^ Composite outcome of acute coronary syndrome, hospitalizations for heart failure, stroke, and cardiovascular death. Includes unstable angina and myocardial infarction.

**Figure 2.**
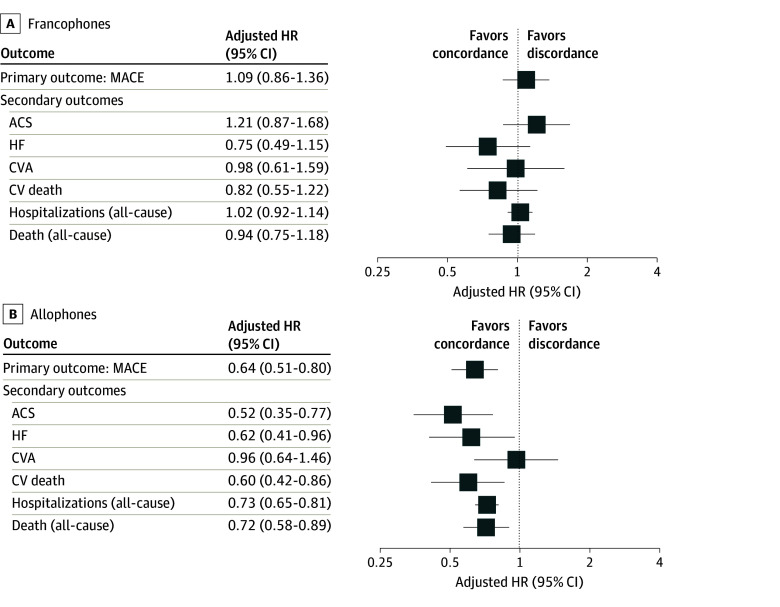
Risk of Cardiovascular (CV) Outcomes and Health Care Use for Patients Receiving Language-Concordant Care Compared With Patients Receiving Language-Discordant Care, Stratified by Nonanglophone Linguistic Group^a^ ACS indicates acute coronary syndrome; CVA, cerebrovascular accident; HF, heart failure; HR, hazard ratio; MACE, major adverse cardiovascular event. ^a^Adjusted HRs could not be reported for Indigenous-speaking patients due to risk of identifying individual respondents with small sample size (n <15).

In the adjusted models (Figure 2), there was no statistically significant difference in the risk of MACEs when comparing patients in the francophone group who received language-concordant care with those in the francophone-group who received language-discordant care (HR, 1.09; 95% CI, 0.86-1.36). Compared with patients in the allophone group who received language-discordant care, those in the allophone group who received language-concordant care were 36% less likely to experience MACEs (HR, 0.64; 95% CI, 0.51-0.80).

### Secondary Outcomes and Subgroup Analyses

For patients in the francophone group, the comparisons between patient-physician language concordance and all secondary outcomes were not statistically significant (Figure 2A). When considering secondary outcomes experienced by patients in the allophone group, we found that patient-physician language concordance was associated with a lower hazard of acute coronary syndrome hospitalization (HR, 0.52; 95% CI, 0.35-0.77), heart failure hospitalization (HR, 0.62; 95% CI, 0.41-0.96), and cardiovascular death (HR, 0.60; 95% CI, 0.42-0.86). There was no association between language concordance and stroke hospitalization in the allophone group (HR, 0.96; 95% CI, 0.64-1.46). The risk of all-cause hospitalization was 27% (HR, 0.73; 95% CI, 0.65-0.81) lower and all-cause mortality was 28% (HR, 0.72; 95% CI, 0.58-0.89) lower in patients in the allophone group who received language-concordant care compared with those in the allophone group who received language-discordant care (Figure 2B).

There were no statistically significant interactions between patient-physician language concordance and prespecified subgroups of interest for the primary outcome (eFigure 1 and eFigure 2 in Supplement 1). In particular, there was no evidence of statistically significant heterogeneity when considering the interaction between patient-physician language concordance and either age (*P* = .69 for the francophone cohort and *P* = .13 for the allophone cohort) or sex (*P* = .21 for the francophone cohort and *P* = .83 for the allophone cohort). Similarly, the interaction between patient-physician language concordance and self-reported knowledge of English was not statistically significant for either linguistic group (*P* = .27 for the francophone cohort and *P* = .84 for the allophone cohort).

## Discussion

In this retrospective national cohort study of Canadians with hypertension, we found that individuals in the allophone group who spoke to their regular physician in their primary home language had a 36% reduction in the relative risk of MACEs compared with those in the allophone group who spoke to their regular physician in a language other than their primary home language. We hypothesize that a combination of cultural and language factors resulting in lower quality of health care services delivered to patients receiving language-discordant care may have a role in the results of this study. While cultural and language factors may impact outcomes through various mechanisms, we propose that patient-physician language concordance is important for comprehensive history taking and establishment of patient-physician trust, which in turn facilitates accurate and timely diagnosis and, ultimately, initiation of effective treatments. This is supported by a growing body of evidence reporting that patient-physician language concordance is positively associated with medical comprehension and understanding, receipt of health counseling (eg, diet, exercise), adherence to follow-up appointments, and overall patient satisfaction.^15,16,17,18^ Furthermore, studies of non–English-speaking patients with diabetes have observed that patient-physician language concordance is associated with improvements in surrogate end points for cardiovascular outcomes, including blood pressure, glycemic, and low-density lipoprotein cholesterol level control,^19,20,21^ as well as a reduction in emergency department visits related to diabetes.^38^

For individuals in the francophone group, the comparison between patient-physician language concordance and cardiovascular outcomes was not statistically significant. We believe that these null findings can be explained by 2 phenomena. First, there exist many policies at the federal and provincial/territorial levels that guarantee access to government services (including health care services) in both official languages.^14^ The fact that more patients in the francophone than allophone group received language-concordant care in our study (68.6% vs 34.1%) suggests that many of these policies are effective. A report commissioned by Health Canada found that approximately three-quarters of French-speaking Canadians living outside of Quebec felt that it was important to receive care in French.^39^ Thus, it is possible that a small minority of patients in the francophone group who identified as having received language-discordant care were in fact receiving care in their preferred language (English), either because they are equally comfortable in English or French or because they prefer to communicate in English in professional settings (eg, due to education and employment opportunities being preferentially available in English). Such misclassification would bias the results toward the null. Next, even though self-reported English proficiency was similar among individuals in the francophone (77.8%) and allophone (80.4%) groups in our study, it is likely that those in the francophone group have greater functional bilingualism than those in the allophone group. Most of the individuals in the allophone group in our study (90.0%) immigrated to Canada, which means that some may have learned English during late childhood or during adulthood, after the so-called critical period for acquisition of native-like proficiency.^40^

Patients who spoke an Indigenous language at home were much less likely to have a regular physician compared with patients who spoke a non-Indigenous language at home. These findings are consistent with those of prior studies that have attributed disparities in access to primary care to geographic location, socioeconomic conditions, and racial discrimination resulting in a lack of trust in the health care system and health care professionals.^41,42^ We also found that fewer patients who spoke an Indigenous language at home received care in that same language, compared with patients who spoke a non-Indigenous language at home. This disparity is likely explained, at least in part, by colonial policies and institutional/systemic racism experienced by Indigenous populations in Canada.^43,44^ The cultural genocide related to the Indian Residential School system robbed many Indigenous populations of their traditional languages. Furthermore, many Indigenous physicians have subsequently lost the ability to speak their traditional languages due to forced assimilation practices, furthering institutional/system racism and mistrust.

### Limitations

This study has some limitations. First, we assumed that respondents would prefer to receive care in the language that they most commonly speak at home. While this assumption may not apply to all respondents, a previous study reported that, in home care and long-term care settings in Ontario, Canada, data collected on a patient’s preferred language had the strongest agreement with the languages spoken most often at home compared with other language variables collected in the CCHS.^45,46^ Furthermore, we excluded respondents who reported speaking multiple languages at home if two or more of their languages corresponded to different linguistic groups (eg, English, French, Indigenous, allophone), since these respondents could not be confidently assigned to one of the mutually exclusive linguistic groups defined in this study. Respondents were only able to submit a single answer to the question asking them to indicate the language spoken with their regular physicians, and it is unclear how respondents receiving care from multiple physicians with different linguistic abilities would answer this question. Next, we do not have information regarding the use of interpretation services. Despite legislation mandating the offer of interpreter services to patients who are unable to receive care in their preferred language, professional interpreter services remain underused in the Canadian health care system,^47,48^ especially among patients who are able to conduct a conversation in English (77.8% of individuals in the francophone group 80.4% of those in the allophone group in this study).

Baseline covariates in this study were self-reported, which means that the results of our analyses may have been subject to self-report bias. However, validation studies have observed that self-reported health status (including hypertension) has good agreement with chronic disease information collected from administrative data.^49,50^ Furthermore, since CCHS data are collected independently from outcomes, misclassification of language variables and baseline covariates should be nondifferential. In addition, the outcomes were identified using objective definitions from administrative data, which reduces the potential for bias due to measurement error. Next, despite adjustment for a wide range of potential confounders, the possibility of unmeasured confounding due to data not being captured for all potential cardiovascular risk factors (eg, dyslipidemia or family history of cardiovascular disease) remains. Finally, the regression analyses were limited to respondents who had a regular physician, which may have preferentially excluded minority language communities who face barriers to accessing primary care.^51,52^ Since the objective of the study was to assess patient-physician language concordance by comparing patients who received language-concordant care with patients who received language-discordant care, this is unlikely to have biased the estimates obtained from regression analyses but did result in smaller sample sizes.

## Conclusions

In this retrospective cohort study, we found large disparities in both access to language-concordant care and cardiovascular outcomes among patients with hypertension living in minority language communities throughout Canada. The results of this study suggest that optimizing the delivery of language-concordant care could result in significant decreases in negative cardiovascular outcomes and excess health care use at the population level. This highlights the importance of advocating for equitable access to medical education for minority language communities to ensure that the linguistic diversity of health care professionals matches that of the patients in their community. In addition, we recommend that data about the languages spoken by patients and health care professionals be systematically collected so that health care systems can implement strategies to match patients to health care professionals who have proficiency in their preferred language.

## Notes

Supplement 1. **eTable 1.** Official Languages by Territory
eReferences 1
**eMethods.** Description of Sampling Frame, Survey Design, and Survey Weights for the CCHS
eReferences 2
**eTable 2.** Language Spoken Most Often at Home
eReferences 3
**eTable 3.** Definitions of Outcomes With Corresponding ICD-10-CA Codes **eTable 4.** Multiple Imputation
eReferences 4
**eTable 5.** Indigenous Languages Represented in This Study **eTable 6.** Allophones Languages Represented in This Study **eTable 7.** Baseline Characteristics of Francophones With a Regular Medical Doctor, Stratified by Patient-Physician Language Concordance **eTable 8.** Baseline Characteristics of Allophones With a Regular Medical Doctor, Stratified by Patient-Physician Language Concordance **eFigure 1.** Subgroup Analyses for Francophones **eFigure 2.** Subgroup Analyses for Allophones

Supplement 2.
Data Sharing Statement
