# Supplementary material for: Patient-Physician Language Concordance and Cardiovascular Outcomes Among Patients With Hypertension
Source: JAMA Netw Open. 2025 Feb 19;8(2):e2460551. doi: 10.1001/jamanetworkopen.2024.60551 (PMC11840650; doi:10.1001/jamanetworkopen.2024.60551)
Supplement: Supplement 1. — eTable 1. Official Languages by Territory eReferences 1 eMethods. Description of Sampling Frame, Survey Design, and Survey Weights for the CCHS eReferences 2 eTable 2. Language Spoken Most Often at Home eReferences 3 eTable 3. Definitions of Outcomes With Corresponding ICD-10-CA Codes eTable 4. Multiple Imputation eReferences 4 eTable 5. Indigenous Languages Represented in This Study eTable 6. Allophones Languages Represented in This Study eTable 7. Baseline Characteristics of Francophones With a Regular Medical Doctor, Stratified by Patient-Physician Language Concordance eTable 8. Baseline Characteristics of Allophones With a Regular Medical Doctor, Stratified by Patient-Physician Language Concordance eFigure 1. Subgroup Analyses for Francophones eFigure 2. Subgroup Analyses for Allophones [file jamanetwopen-e2460551-s001.pdf]

Supplementary Online Content

Reaume M, Labossière MN, Batista R, et al. Patient-physician language concordance and cardiovascular outcomes among patients with hypertension. *JAMA Netw Open*. 2025;8(2):e2460551. doi:10.1001/jamanetworkopen.2024.60551

eTable 1. Official languages by Territory

eReferences 1

eMethods. Description of Sampling Frame, Survey Design, and Survey Weights for the CCHS

eReferences 2

eTable 2. Language Spoken Most Often at Home

eReferences 3

eTable 3. Definitions of Outcomes With Corresponding *ICD-10-CA* Codes

eTable 4. Multiple Imputation

eReferences 4

eTable 5. Indigenous Languages Represented in This Study

eTable 6. Allophones Languages Represented in This Study

eTable 7. Baseline Characteristics of Francophones With a Regular Medical Doctor, Stratified by Patient-Physician Language Concordance

eTable 8. Baseline Characteristics of Allophones With a Regular Medical Doctor, Stratified by Patient-Physician Language Concordance

eFigure 1. Subgroup Analyses for Francophones

eFigure 2. Subgroup Analyses for Allophones

This supplementary material has been provided by the authors to give readers additional information about their work.

© 2025 Reaume M et al. *JAMA Network Open.*

eTable 1. Official languages by territory.

Territory	Official languages
Yukon	English, French
Northwest Territories	Dene Kədə́, Dëne Sųłıné, Dene Zhatıé, Dinjii Zhu' Ginjik, English, French, Inuinnaqtun, Inuktitut, Inuvialuktun, nēhiyawēwin, Tłįchǫ
Nunavut	English, French, Inuinnaqtun, Inuktitut, Inuvialuktun

eReferences 1

1. Government of Northwest Territories. Official Languages. https://www.ece.gov.nt.ca/en/official-languages. Accessed June 11, 2024.
2. Government of Yukon. Diverse Territory. https://yukon.ca/en/diverse-territory. Accessed June 11, 2024.
3. Office of the Languages Commissionner of Nunavut. Your Linguistic Rights. https://langcom.nu.ca/investigation-process/your-linguistic-rights. Accessed June 11, 2024.
4. Statistics Canada. While English and French are still the main languages spoken in Canada, the country's linguistic diversity continues to grow. https://www150.statcan.gc.ca/n1/daily-quotidien/220817/dq220817a-eng.htm. Accessed February 24, 2024.

© 2025 Reaume M et al. *JAMA Network Open.*

eMethods. Description of sampling frame, survey design, and survey weights for the CCHS.

The CCHS includes individuals 12 years of age or older who live in a private dwelling in any Canadian province or territory, with the exception of those: 1) living in the Quebec health regions of Région du Nunavik and Région des Terres-Cries-de-la-Baie-James, 2) living on reserves and other Aboriginal settlements in the provinces, 3) who are institutionalized, 4) who are members of the Canadian forces. Individuals excluded from the CCHS sampling frame represent approximately 3% of the Canadian population. Statistics Canada selects respondents from two different sampling frames: an area frame and a list frame of telephone numbers. To ensure that sufficiently large samples are collected from each province and health region, a stratified multi-stage cluster sampling scheme is employed, with sample size allocated to provinces and health regions. Clusters of households within each health region are selected using probability proportional to size, then individual households are selected using systematic sampling. Finally, one or two individuals are selected from each household according to pre-determined age-based selection probabilities.

In order to compute summary statistics (e.g., means, proportions) at the population level, Statistics Canada releases sampling weights, which are defined as the inverse of the probability of selection, and which provide a measure of how many people in the population are represented by each respondent. Summary statistics are obtained by taking the sum of each respondent's individual statistic multiplied by their corresponding weight, and then dividing by the sum of the respondents' weights.

As the objective of this study was to determine the association between patient-physician language concordance and cardiovascular outcomes among a nationally representative cohort of patients with self-reported hypertension, we performed model-based analyses that did not incorporate sampling weights.[3-5] Unadjusted patient characteristics and outcomes are reported for descriptive purposes only, and should not be used to make inferences regarding summary statistics (e.g., means, proportions) at the population level.

All respondents are provided with the option of completing the interview in either English or French. However, Statistics Canada recruits interviewers who speak languages other than English or French, and encourages its regional offices to transfer respondents whose preferred language is a language other than English or French to interviewers who have the language competency/proficiency necessary to administer the interview in the respondents' preferred language.

eReferences 2

1. Béland Y, Dale V, Dufour J, Hamel M. The Canadian Community Health Survey: Building on the Success from the Past. In: *Proceedings of the Survey Research Methods Section*. American Statistical Association; 2005.
2. Statistics Canada. Canadian Community Health Survey - Annual Component (CCHS). https://www23.statcan.gc.ca/imdb/p2SV.pl?Function=getSurvey&SDDS=3226. Accessed February 24, 2024.

© 2025 Reaume M et al. *JAMA Network Open.*

3. Little RJA. To Model or Not To Model? Competing Modes of Inference for Finite Population Sampling. *J Am*. 2004;99(466):546-556. doi:10.1198/016214504000000467

4. Lee ES, Forthofer RN. *Analyzing Complex Survey Data*. Thousand Oaks, CA: SAGE Publications Ltd; 2006.

5. Lumley T. *Complex Surveys: A Guide to Analysis Using R*. Hoboken, NJ: John Wiley & Sons; 2010.

© 2025 Reaume M et al. *JAMA Network Open.*

eTable 2. Language spoken most often at home

Below is a table describing the individual languages collected for the variable "language spoken most often at home" in the CCHS from 2003 to 2014. The CCHS underwent two important methodological changes affecting the variable "language spoken most often at home" during this time frame:

- Prior to 2007, respondents were asked to report the single language that they speak most often at home. Since 2007, respondents who speak more than one language at home have been able to provide multiple responses to this question.
- Prior to 2011, data were only captured for 1 Indigenous language and 19 Allophone languages. Respondents who did not speak English, French, or one of these 20 languages were coded as speaking "other". In 2011, several additional Indigenous and Allophone languages were added to the list of answer choices. As such, considerably fewer respondents were coded as speaking "other" from 2011 to 2014.

Year	Anglophones	Francophones	Indigenous	Allophones
2003-2010	English	French	Cree	Arabic, Chinese, Dutch, German, Greek, Hindi, Hungarian, Italian, Korean, Persian (Farsi), Polish, Portuguese, Punjabi, Russian, Spanish, Tagalog (Pilipino), Tamil, Ukrainian, Vietnamese
2011-2014	English	French	Aboriginal languages, Algonquian languages, Athapaskan languages, Atikamekw, Blackfoot, Carrier, Chilcotin, Chipewyan, Cree, Dene, Dogrib, Gitksan, Haida, Inuinnaqtun, Inuktitut, Kutchin-Gwich'in (Loucheux), Kutenai, Malecite, Mi'kmaq, Mohawk, Montagnais-Naskapi, Nisga'a, Nootka, North Slave (Hare), Ojibway, Oji-Cree, Salish languages, Shuswap, Siouan languages (Dakota/Sioux), South Slave, Tlingit, Thompson (Ntlakapamux), Tsimshian, Wakashan languages	Afro-Asiatic languages, Akan (Twi), Amharic, Arabic, Armenian, Austro-Asiatic languages, Azerbaijani, Baltic languages, Bantu languages, Berber languages (Kabyle), Belarusan (Byelorussian), Bengali, Bisayan languages, Bosnian, Bulgarian, Cantonese, Celtic languages, Chaochow (Teochow), Chinese languages, Creoles, Croatian, Cushitic languages, Czech, Danish, Dravidian languages, Dutch, Edo, Estonian, Finnish, Finno-Ugric languages, Flemish, Frisian, Fukien, Gaelic languages, German, Germanic languages, Greek, Gujarati, Hakka, Hebrew, Hindi, Hungarian, Icelandic, Igbo, Ilocano, Indo-Aryan languages, Indo-Iranian language, Iranian languages, Italian, Japanese, Kannada, Khmer (Cambodian), Konkani, Korean, Kurdish, Lao, Latvian, Lingala, Lithuanian, Macedonian, Malay, Malayalam, Malayo-Polynesian languages, Maltese, Mandarin, Marathi, Niger-Congo languages, Norwegian, Oromo, Panjabi (Punjabi), Pampango, Pashto, Persian (Farsi), Polish, Portuguese, Romance languages, Romanian, Russian, Rwanda (Kinyarwanda), Scandinavian languages, Semitic languages, Serbian, Serbo-Croatian, Shanghainese, Shona, Sindhi, Sinhala (Sinhalese), Sino-tibetan languages, Slavic languages, Slovak, Slovenian, Somali, Spanish, Swahili, Swedish, Tagalog (Filipino/Pilipino), Tai languages, Taiwanese, Tamil, Telugu, Thai, Tibetan languages, Tigrigna, Turkic languages, Turkish, Ukrainian, Urdu, Vietnamese, Welsh, Wolof, Yiddish

eReferences 3

1. Statistics Canada. Canadian Community Health Survey - Annual Component (CCHS). https://www23.statcan.gc.ca/imdb/p2SV.pl?Function=getSurvey&SDDS=3226. Accessed February 24, 2024.

© 2025 Reaume M et al. *JAMA Network Open.*

eTable 3. Definitions of outcomes with corresponding ICD-10-CA codes.

Outcome	ICD-10 code (most responsible diagnosis)	Data Source
MACE: first occurrence of acute coronary hospitalization, heart failure hospitalization, stroke hospitalization, or cardiovascular death	See below	DAD and CVSD
Acute coronary syndrome hospitalization	Includes both unstable angina and myocardial infarction (see below)	DAD
Unstable angina hospitalization	I20, I24.0	DAD
Myocardial infarction hospitalization	I21, I22	DAD
Heart failure hospitalization	I11.0, I13, I50, I97.1, I132	DAD
Stroke hospitalization	I60-I64	DAD
Cardiovascular death	I00-I99	CVSD
CVSD = Canadian Vital Statistics Database; DAD = Discharge Abstract Database		

© 2025 Reaume M et al. *JAMA Network Open.*

eTable 4. Multiple imputation.

We performed multiple imputation by chained equation,[1] with number of imputed datasets and number of iterations both set to 10. The models used to estimate responses on missing categorical variables included three auxiliary variables (patient's primary home language, patient's knowledge of English, and the language spoken with the patient's regular medical doctor), all model variables (including exposure and covariates), the censoring variable, and the time-to-event variable (which was transformed to the cumulative survival function using the Nelson-Aalen estimator).[2] We used binary logistic regression for binary covariates, proportional odds model for ordered categorical covariates, and multinomial logistic regression for unordered categorical variables.[1] The following table contains list of covariates included in the imputation model, with corresponding variable type, missing data, and imputation model.

Covariate	Variable Type	Missing Data – n (%)[a]	Imputation Model
Age	Continuous	0 (0%)	N/A
Sex	Binary	0 (0%)	N/A
Marital Status	Binary	116 (0.1%)	Binary logistic regression
Education	Categorical	1,177 (0.9%)	Proportional odds model
Household Income Quintile	Categorical	10,837 (8.7%)	Proportional odds model
Geographic region	Categorical	0 (0%)	N/A
Urban/Rural Residence	Binary	0 (0%)	N/A
Immigrant	Binary	224 (0.2%)	Binary logistic regression
Diabetes	Binary	113 (0.1%)	Binary logistic regression
History of Heart Disease	Binary	631 (0.5%)	Binary logistic regression
History of Stroke	Binary	176 (0.1%)	Binary logistic regression
Obesity (by BMI)	Categorical	6205 (5.0%)	Proportional odds model
Smoking	Categorical	332 (0.3%)	Multinomial logistic regression

[a] Percentage refers to the number of missing data across all linguistic groups divided by the study sample size (n=124,583).

eReferences 4

1. Azur MJ, Stuart EA, Frangakis C, Leaf PJ. Multiple imputation by chained equations: what is it and how does it work? *Int J Methods Psychiatr Res*. 2011;20(1):40-49. doi:10.1002/mpr.329
2. White IR, Royston P. Imputing missing covariate values for the Cox model. *Stat Med*. 2009;28(1):1982–1998. doi:10.1002/sim.3618

© 2025 Reaume M et al. *JAMA Network Open.*

eTable 5. Indigenous languages represented in this study.

Language	N (%)
Cree	85 (26.2%)
Inuit (including Inuinnaqtun and Inuktitut)	49 (15.1%)
Other (could not be released due to small sample sizes[a])	23 (7.1%)
Other (reported as such in CCHS)	168 (51.7%)
**Total**	**325 (100.0%)**

[a] Includes Chipewyan, Dene, Dogrib, Mohawk, Nisga'a, South Slave.

© 2025 Reaume M et al. *JAMA Network Open.*

eTable 6. Allophones languages represented in this study.

Language	N (%)
Amharic	17 (0.3%)
Arabic	98 (1.9%)
Chinese languages	770 (14.7%)
Dutch	108 (2.1%)
Gaelic languages	17 (0.3%)
German	507 (9.7%)
Greek	67 (1.3%)
Gujarati	17 (0.3%)
Hindi	58 (1.1%)
Hungarian	85 (1.6%)
Italian	559 (10.7%)
Korean	72 (1.4%)
Persian (Farsi)	73 (1.4%)
Polish	272 (5.2%)
Portuguese	312 (6.0%)
Punjabi	268 (5.1%)
Russian	128 (2.4%)
Sinhala (Sinhalese)	18 (0.3%)
Spanish	193 (3.7%)
Tagalog (Filipino or Pilipino)	296 (5.7%)
Tamil	64 (1.2%)
Ukrainian	127 (2.4%)
Urdu	57 (1.1%)
Vietnamese	51 (1.0%)
Other (could not be released due to small sample sizes[a])	275 (5.3%)
Other (reported as such in CCHS)	720 (13.8%)
**Total**	**5,229 (100.0%)**

[a] Includes Afro-Asiatic languages, Akan (Twi), Armenian, Baltic languages, Belarusan (Byelorussian), Bengali, Berber languages (Kabyle), Bisayan languages, Bosnian, Bulgarian, Cambodian / Khmer, Celtic languages, Creoles, Croatian, Cushitic languages, Czech, Danish, Dravidian languages, Edo, Estonian, Finnish, Flemish, Frisian, Germanic languages, Hebrew, Icelandic, Ilocano, Indo-Aryan languages, Indo-Iranian languages, Iranian languages, Japanese, Latvian, Lithuanian, Macedonian, Malay, Malayalam, Malayo-Polynesian languages, Maltese, Marathi, Norwegian, Oromo, Pashto, Romance languages, Romanian, Rwanda (Kinyarwanda), Serbian, Serbo-Croatian, Sindhi, Slovak, Slovenian, Somali, Swahili, Swedish, Telugu, Thai, Turkish, Welsh.

eTable 7. Baseline characteristics of Francophones with a regular medical doctor, stratified by patient-physician language concordance.

Baseline Characteristics	Francophones (N = 4,566)		
	Language-Concordant Care (N = 3,134)	Language-Discordant Care (N = 1,432)	P-Value*
**Sociodemographic characteristics**			
**Age – mean +/- SD**	63.4 +/- 14.0	64.4 +/- 13.5	0.02
**Age – n (%)**			0.03
Age < 60	1,156 (36.9)	472 (33.0)	
Age 60-75	1,266 (40.4)	621 (43.4)	
Age >= 75	712 (22.7)	339 (23.7)	
**Sex – n (%)**			0.16
Female	1,923 (61.4)	847 (59.1)	
Male	1,211 (38.6)	585 (40.9)	
**Marital Status – n (%)**			0.41
Single	1,323 (42.2)	585 (40.9)	
Married or common-law	1,808 (57.7)	843 (58.9)	
Missing	3 (0.1)	4 (0.3)	
**Education – n (%)**			< 0.01
Less than high school	1,485 (47.4)	598 (41.8)	
High school graduate	460 (14.7)	211 (14.7)	
Post-secondary graduate	1,151 (36.7)	606 (42.3)	
Missing	38 (1.2)	17 (1.2)	
**Household Income Quintile – n (%)**			0.20
1 (lowest)	993 (31.7)	415 (29.0)	
2	763 (24.3)	307 (21.4)	
3	473 (15.1)	221 (15.4)	
4	400 (12.8)	196 (13.7)	
5 (highest)	309 (9.9)	153 (10.7)	
Missing	196 (6.3)	140 (9.8)	
**Geographic Region – n (%)**			< 0.01
Western provinces/territories	63 (2.0)	240 (16.8)	
Ontario	821 (26.2)	698 (48.7)	
Maritime provinces	2,250 (71.8)	494 (34.5)	
**Urban/Rural Residence – n (%)**			< 0.01
Rural	1,576 (50.3)	528 (36.9)	
Urban	1,558 (49.7)	904 (63.1)	
**Immigrant – n (%)**			< 0.01
Yes	34 (1.1)	45 (3.1)	
No	3,098 (98.9)	1,387 (96.9)	
Missing	2 (0.1)	0 (0.0)	
**Comorbidities**			
**Diabetes – n (%)**	594 (19.0)	293 (20.5)	0.24
**Heart disease or Stroke – n (%)**	617 (19.7)	298 (20.8)	0.39
**Obesity (by BMI) – n (%)**			0.34
Normal (BMI < 25.0)	869 (27.7)	427 (29.8)	
Overweight (BMI 25.0-29.9)	1,191 (38.0)	523 (36.5)	
Obesity (BMI >= 30.0)	935 (29.8)	421 (29.4)	
Missing	139 (4.4)	61 (4.3)	
**Smoking – n (%)**			0.53
Current	489 (15.6)	237 (16.6)	
Former	1,676 (53.5)	772 (53.9)	
Never	962 (30.7)	419 (29.3)	
Missing	7 (0.2)	4 (0.3)	

BMI = Body Mass Index; SD = Standard Deviation
* Baseline characteristics were compared using chi-squared test for categorical variables and t-test for continuous variables. Missing values were excluded in tests for statistical significance.

© 2025 Reaume M et al. *JAMA Network Open.*

eTable 8. Baseline characteristics of Allophones with a regular medical doctor, stratified by patient-physician language concordance.

Baseline Characteristics	Allophones (N = 5,013)		
	**Language-Concordant Care** (N = 1,708)	**Language-Discordant Care** (N = 3,305)	**P-Value***
**Sociodemographic characteristics**			
**Age – mean +/- SD**	65.0 +/- 14.5	63.9 +/- 14.9	0.02
**Age – n (%)**			0.47
Age < 60	565 (33.1%)	1,113 (33.7%)	
Age 60-75	660 (38.6%)	1,311 (39.7%)	
Age >= 75	483 (28.3%)	881 (26.7%)	
**Sex – n (%)**			0.02
Female	1,022 (59.8)	1,787 (54.1)	
Male	686 (40.2)	1,518 (45.9)	
**Marital Status – n (%)**			< 0.01
Single	557 (32.6)	942 (28.5)	
Married or common-law	1,150 (67.3)	2,361 (71.4)	
Missing	1 (0.1)	2 (0.1)	
**Education – n (%)**			< 0.01
Less than high school	739 (43.3)	1,242 (37.6)	
High school graduate	320 (18.7)	519 (15.7)	
Post-secondary graduate	628 (36.8)	1,496 (45.3)	
Missing	21 (1.2)	48 (1.5)	
**Household Income Quintile – n (%)**			< 0.01
1 (lowest)	821 (48.1)	1,173 (35.5)	
2	313 (18.3)	767 (23.2)	
3	195 (11.4)	452 (13.7)	
4	120 (7.0)	325 (9.8)	
5 (highest)	48 (2.8)	225 (6.8)	
Missing	211 (12.4)	363 (11.0)	
**Geographic Region – n (%)**			0.26
Western provinces/territories	719 (42.1)	1,337 (40.5)	
Ontario and maritime provinces	989 (57.9)	1,968 (59.5)	
**Urban/Rural Residence – n (%)**			< 0.01
Rural	30 (1.8)	401 (12.1)	
Urban	1,678 (98.2)	2,904 (87.9)	
**Immigrant – n (%)**			< 0.01
Yes	1,653 (96.8)	2,868 (86.8)	
No	40 (2.3)	411 (12.4)	
Missing	15 (0.9)	26 (0.8)	
**Comorbidities**			
**Diabetes – n (%)**	389 (22.8)	703 (21.3)	0.23
**Heart disease or Stroke – n (%)**	315 (18.4)	632 (19.1)	0.54
**Obesity (by BMI) – n (%)**			< 0.01
Normal (BMI < 25.0)	706 (41.3)	982 (29.7)	
Overweight (BMI 25.0-29.9)	559 (32.7)	1,315 (39.8)	
Obesity (BMI >= 30.0)	258 (15.1)	777 (23.5)	
Missing	185 (10.8)	231 (7.0)	
**Smoking – n (%)**			< 0.01
Current	125 (7.3)	295 (8.9)	
Former	424 (24.8)	1,191 (36.0)	
Never	1,156 (67.7)	1,809 (54.7)	
Missing	3 (0.2)	10 (0.3)	

BMI = Body Mass Index; SD = Standard Deviation
* Baseline characteristics were compared using chi-squared test for categorical variables and t-test for continuous variables. Missing values were excluded in tests for statistical significance.

© 2025 Reaume M et al. *JAMA Network Open.*

eFigure 1. Subgroup analyses for Francophones.

Subgroup	No. of Patients	Adjusted Hazard Ratio (95% CI)	P Value for Interaction
All patients	4566	1.09 (0.86-1.36)	
Age			0.69
Age < 60	1628	0.88 (0.49-1.58)	
Age 60-75	1887	1.18 (0.83-1.68)	
Age >= 75	1051	1.07 (0.78-1.46)	
Sex			0.21
Female	2770	1.26 (0.91-1.73)	
Male	1796	0.95 (0.70-1.29)	
Marital Status			0.73
Single	1908	1.05 (0.77-1.42)	
Married or common-law	2651	1.13 (0.82-1.54)	
Education			0.70
Less than high school education	2083	1.16 (0.87-1.55)	
High school graduate	671	1.10 (0.50-2.44)	
Post-secondary graduate	1757	0.95 (0.64-1.40)	
Household Income Quintile			0.87
1 (lowest)	1408	1.01 (0.71-1.43)	
2	1070	1.24 (0.80-1.94)	
3	694	0.98 (0.54-1.75)	
4 or 5 (highest)	1058	1.10 (0.63-1.93)	
Geographic Region			0.44
Western provinces and territories	303	0.84 (0.35-2.02)	
Ontario	1519	0.96 (0.69-1.34)	
Maritime provinces	2744	1.29 (0.90-1.85)	
Urban/Rural Residence			0.58
Rural	2104	1.17 (0.82-1.68)	
Urban	2462	1.03 (0.78-1.37)	
Immigrant			0.26
No	4485	1.06 (0.84-1.34)	
Yes	79	2.44 (0.60-9.82)	
Knowledge of English (self-reported)			0.27
No	1016	0.70 (0.34-1.46)	
Yes	3549	1.09 (0.86-1.40)	
History of heart disease or stroke			0.57
No	3639	1.01 (0.75-1.36)	
Yes	915	1.15 (0.83-1.60)	

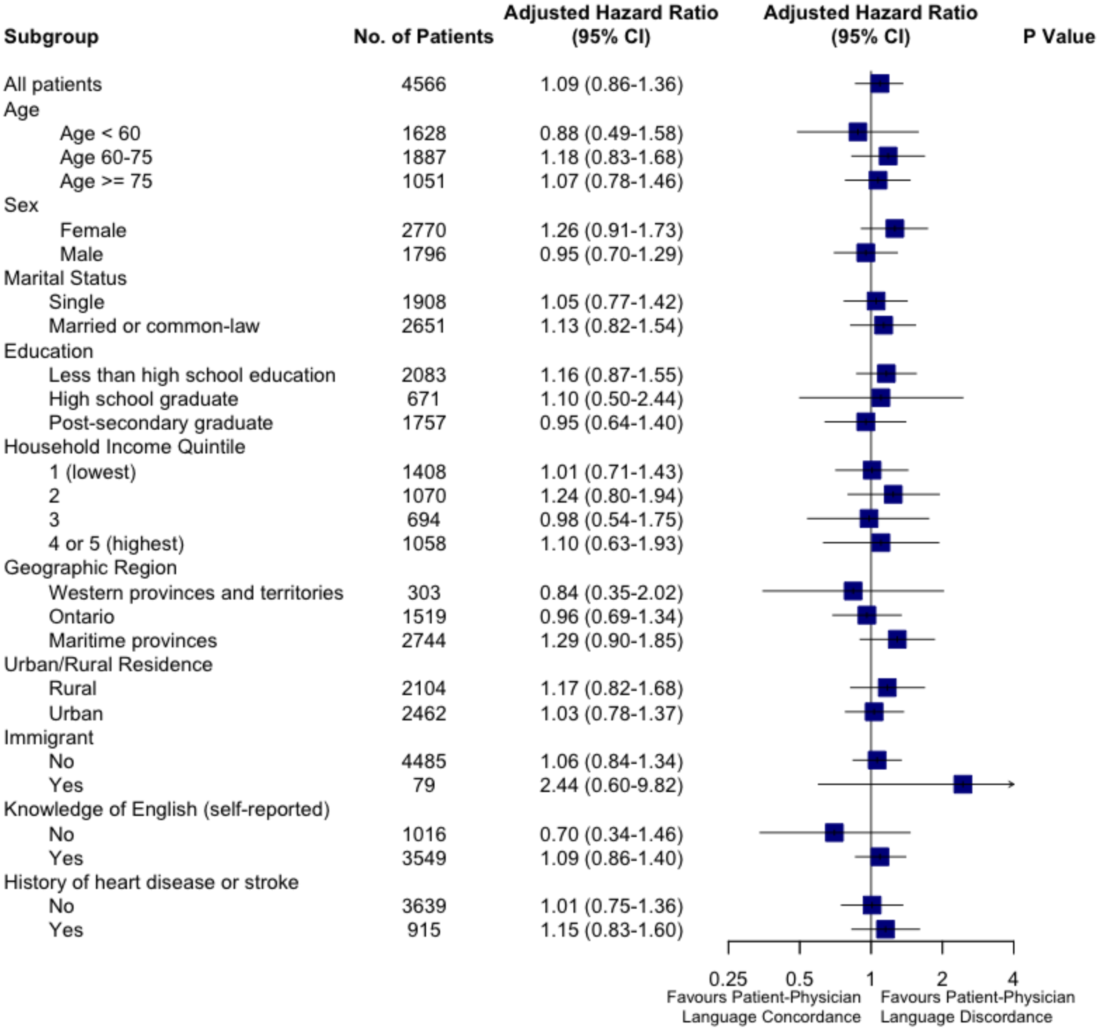

© 2025 Reaume M et al. *JAMA Network Open.*

eFigure 2. Subgroup analyses for Allophones.

Subgroup	No. of Patients	Adjusted Hazard Ratio (95% CI)	P Value for Interaction
All patients	5013	0.64 (0.51-0.80)	
Age			0.13
Age < 60	1678	0.64 (0.29-1.43)	
Age 60-75	1971	0.92 (0.62-1.36)	
Age >= 75	1364	0.55 (0.41-0.73)	
Sex			0.83
Female	2809	0.62 (0.46-0.84)	
Male	2204	0.65 (0.47-0.91)	
Marital Status			0.52
Single	1499	0.59 (0.42-0.82)	
Married or common-law	3511	0.68 (0.50-0.92)	
Education			0.74
Less than high school education	1981	0.68 (0.51-0.91)	
High school graduate	839	0.64 (0.35-1.18)	
Post-secondary graduate	2124	0.55 (0.35-0.86)	
Household Income Quintile			0.41
1 (lowest)	1994	0.69 (0.51-0.93)	
2	1080	0.82 (0.51-1.34)	
3	647	0.38 (0.17-0.87)	
4 or 5 (highest)	718	0.49 (0.20-1.19)	
Geographic Region			0.69
Western provinces and territories	2056	0.67 (0.47-0.97)	
Ontario and maritime provinces	2957	0.62 (0.46-0.82)	
Urban/Rural Residence			0.35
Rural	431	1.13 (0.35-3.70)	
Urban	4582	0.63 (0.50-0.79)	
Immigrant			0.14
No	451	1.42 (0.50-4.05)	
Yes	4521	0.61 (0.48-0.78)	
Knowledge of English (self-reported)			0.84
No	1001	0.59 (0.39-0.90)	
Yes	4009	0.63 (0.46-0.86)	
History of heart disease or stroke			0.83
No	4038	0.66 (0.48-0.89)	
Yes	947	0.62 (0.45-0.87)	

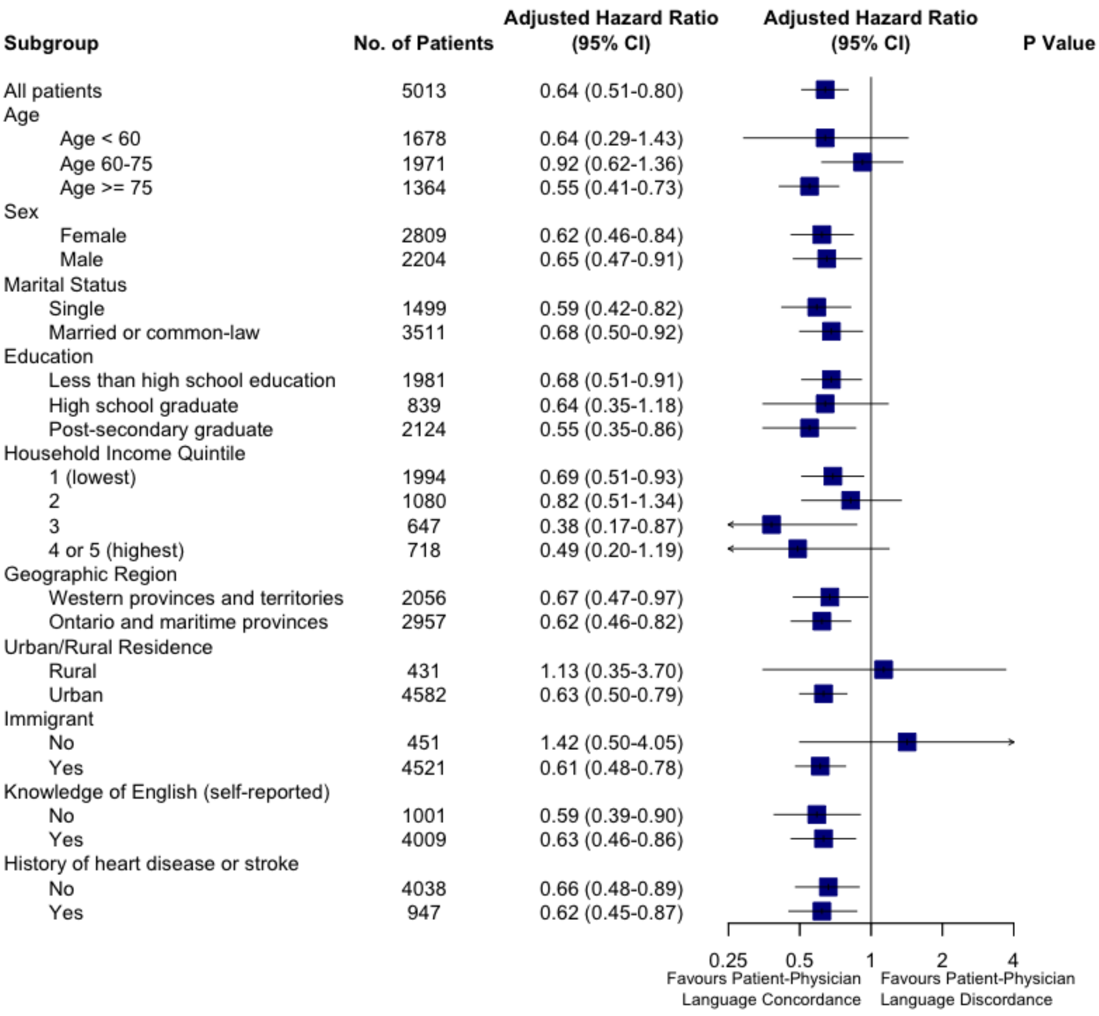

© 2025 Reaume M et al. *JAMA Network Open.*
