# Supplementary material for: Patient-Physician Language Concordance and Cardiovascular Outcomes Among Patients With Hypertension
Source: JAMA Netw Open. 2025 Feb 19;8(2):e2460551. doi: 10.1001/jamanetworkopen.2024.60551 (PMC11840650; doi:10.1001/jamanetworkopen.2024.60551)
Supplement: Supplement 2. — Data Sharing Statement [file jamanetwopen-e2460551-s002.pdf]

Data Sharing Statement

Reaume. Patient-Physician Language Concordance and Cardiovascular Outcomes Among Patients With Hypertension. *JAMA Netw Open*. Published February 19, 2025.
doi:10.1001/jamanetworkopen.2024.60551

Data
Data available: No

Additional Information
Explanation for why data not available: Data used for this study consists primarily of survey data (Canadian Community Health Survey) collected by Statistics Canada. While aggregate or summary data are publicly available via Statistics Canada website, the microdata files containing individual level data are only available at designated Research Data Centres through a mechanism set out by legislation and regulation. As such, requests for data sharing should be directed to Statistics Canada Research Data Centres (https://www.statcan.gc.ca/en/microdata/data-centre) to determine whether researchers meet the criteria to access microdata files.
